# The addition of rituximab to chemotherapy improves overall survival in mantle cell lymphoma—a pooled trials analysis

**DOI:** 10.1007/s00277-023-05385-1

**Published:** 2023-08-08

**Authors:** Luca Fischer, Linmiao Jiang, Joerg Thomas Bittenbring, Kai Huebel, Christian Schmidt, Johannes Duell, Bernd Metzner, Juergen Krauter, Bertram Glass, Andreas Huettmann, Kerstin Schaefer-Eckart, Elisabeth Silkenstedt, Wolfram Klapper, Wolfgang Hiddemann, Michael Unterhalt, Martin Dreyling, Eva Hoster, on behalf of the German Lymphoma Alliance (GLA) and the German Low-Grade Lymphoma Study Group (GLSG)

**Affiliations:** 1 Department of Medicine III, University Hospital, LMU Munich, Munich, Germany; 2 Institute for Medical Information Processing, Biometry and Epidemiology, LMU Munich, Munich, Germany; 3 University of Saarland, University Hospital, Homburg (Saar), Homburg, Germany; 4 University of Cologne, Cologne, Germany; 5 Department of Internal Medicine II, University Hospital Würzburg, University of Würzburg, Würzburg, Germany; 6 University Clinic for Oncology and Hematology, Klinikum Oldenburg, Oldenburg, Germany; 7 Department of Internal Medicine III, Braunschweig Municipal Hospital, Brunswick, Germany; 8 Department of Hematology, HELIOS Klinikum Berlin-Buch, Berlin, Germany; 9 Department of Hematology and Stem Cell Transplantation, West German Cancer Center Essen, University Hospital Essen (AöR), University of Duisburg-Essen, Essen, Germany; 10 Medical Clinic V, Hematology and Oncology, Klinikum Nuernberg, Nuernberg, Germany; 11 Department of Pathology, Hematopathology Section, University Hospital Schleswig-Holstein, Christian-Albrecht-University of Kiel, Kiel, Germany

**Keywords:** Mantle cell lymphoma, Rituximab, Long-term outcome, Immuno-chemotherapy

## Abstract

Mantle cell lymphoma (MCL) is a distinct subtype of B-cell lymphoma and commonly used induction immunochemotherapies include the anti-CD20 antibody rituximab. However, efficacy data for rituximab regarding overall survival (OS) in first line MCL therapy remain conflicting.

We report long-term outcomes of a pooled trials analysis comparing Cyclophosphamide, Doxorubicine, Vincristine, Prednisone (CHOP) to R-CHOP in MCL to confirm efficacy on failure free survival (FFS) and OS in relevant subgroups. Untreated, adult MCL patients of two prospective trials assigned to CHOP or R-CHOP were included. Primary endpoints were FFS and OS, secondary endpoints included duration of response (DOR), secondary malignancies and OS after relapse. Between 1996 and 2003, 385 MCL patients were assigned to CHOP (201) or R-CHOP (184). After a median follow-up of 13.4 years, the addition of Rituximab significantly improved FFS (1.36 vs. 2.07 years, HR 0.62 (0.50–0.77)), OS (4.84 vs. 5.81 years, HR 0.78 (0.61–0.99)) and DOR (1.48 vs. 2.08 years, HR 0.67 (0.53–0.86)). Furthermore, Rituximab improved survival across different MCL risk groups. In a post-hoc analysis of OS after relapse comparing patients receiving chemotherapy with / without rituximab, rituximab maintained efficacy with a median OS of 3.10 vs. 2.11 years (HR 0.70, 0.54–0.91). The rate of secondary malignancies was 0.5 and 3.9% for hematological and 7 and 8% for non-hematological malignancies for CHOP and R-CHOP patients, respectively. We present mature results of a pooled MCL cohort, demonstrating prolonged FFS, OS and DOR for the combined immuno-chemotherapy, confirming the standard of care in first line treatment.

**Supplementary Information:**

The online version contains supplementary material available at 10.1007/s00277-023-05385-1.

## Introduction

Mantle Cell Lymphoma (MCL) is a rare B-cell Non-Hodgkin Lymphoma accounting for 6–8% of mature B-cell neoplasias with an incidence rate of 1–2/100 000 persons per year [1, 2]. Median age at diagnosis ranges between 60–70 years and with a ratio of about 3–4:1, men are more frequently affected than women [2, 3].

Current therapeutic strategies for advanced stage mantle cell lymphoma involve intensive cytarabine containing immunochemotherapy regimes with consolidating autologous stem cell transplantation (ASCT) for younger, and less intensive immunochemotherapy strategies for older patients, each followed by rituximab maintenance [4]. Several clinical and biological prognostic scores have been proposed to date, with the mantle cell lymphoma international prognostic index (MIPI) including age, performance status, LDH and leucocyte count, and MIPI-c, including Ki-67 proliferation index being the most extensively validated [5–7]. In the last two decades, long-term outcome has substantially improved through intensified induction regimens, now achieving median times to treatment failure of 7–9 years in younger patients [8–10]. In older patients, intensive induction regimens are not feasible and cyclophosphamide, doxorubicine, vincristine and prednisone (CHOP) remains a relevant chemotherapeutic backbone [4, 11].

One of the first randomized GLSG trials (GLSG1996) compared CHOP to MCP (Mitoxantrone, Chlorambucil, Prednisone), with CHOP showing better response rates with less haematological toxicity and better stem cell mobilisation [12]. In 2000, the GLSG started a new trial for induction in advanced stage MCL, GLSG2000, comparing CHOP with R-CHOP [13]. In both trials, post-remission treatment comprised interferon-alpha maintenance (IFN) for patients > 65 years. Younger patients suitable for high dose therapy, were randomized upfront for ASCT or IFN within the first European MCL network trial (MCL-1) [14].

All first line treatment protocols feature anti-CD20 directed monoclonal antibodies, mostly rituximab. Despite its widespread use and integration into current treatment guidelines, evidence for rituximab’s efficacy in MCL during induction therapy regarding overall survival is still conflicting: Rituximab was first shown to be effective in relapsed MCL patients [15]. In untreated patients, rituximab was repeatedly shown to increase response and time to treatment failure (TTF), but only one in three randomized trials demonstrated significant OS improvement. This study used fludarabine cyclophosphamide (FC) as chemotherapeutic backbone and median OS was short compared to the other trials [13, 16, 17]. In a meta-analysis published in 2007, the effect of rituximab added to induction chemotherapy in MCL was significant for OS with a hazard ratio for mortality of 0.60 (95% CI 0.37–0.98), but with a noted heterogeneity among trials and without significance if restricted to the first-line trials (HR 0.78, 95% CI 0.45–1.35) [18]. Thus, the current evidence for rituximab efficacy as part of induction in first-line MCL in terms of the most clinically relevant endpoint OS remains debatable. One important reason is the small patient number, even in the meta-analysis. To achieve adequate statistical power, we pooled and analyzed the long-term follow-up data of all MCL patients treated within the GLSG1996 and GLSG2000 trials with CHOP and R-CHOP to more reliably estimate treatment effects by the addition of rituximab to CHOP with a focus on OS and relevant subgroups.

## Methods

### Study design and patients

This is a pooled individual data analysis of a randomized trial (GLSG2000) and two prospective cohorts embedded in open-label randomized trials (GLSG1996 and GLSG2000). After randomization in GLSG1996 was stopped in 1998 due to superior efficacy of CHOP over MCP, recruitment to the trial remained open until start of GLSG2000 in the year 2000, assigning new patients to CHOP chemotherapy. Similarly, after stop of randomization in July 2002 in GLSG2000 due to superior efficacy of R-CHOP over CHOP, recruitment to the trial remained open until May 2003, assigning new patients to R-CHOP. In both trials, inclusion, treatment, assessments, and documentation of patients registered with fixed treatment allocation after stop of randomization was done according to the respective trial protocol.

Inclusion criteria of GLSG1996 and 2000 trials were similar and have been published in detail previously [12, 13]. In summary, patients ≥ 18 years with confirmed untreated MCL stage III-IV disease were eligible. Patients with an ECOG > 2 not related to lymphoma, heavily reduced organ function, uncontrolled comorbidities, pre-treatment with IFN or cytostatic agents, as well as patients with a history of previous organ or bone marrow transplant were not enrolled. Randomization (CHOP vs. MCP and CHOP vs. R-CHOP) was done centrally and stratified by age and number of IPI risk factors. Patients ≤ 65 years and suitable for ASCT were eligible to enter a second trial, the first MCL network trial, with upfront randomization between ASCT and IFN [14, 19]. Patients > 65 years of age or unfit to receive ASCT were randomized at end of induction to receive standard or intensified IFN treatment.

All patients with histologically confirmed MCL and prospectively assigned to either CHOP or R-CHOP within the GLSG1996 or GLSG2000 trials are included in this pooled analysis. Reference pathology review was performed for all included patients. All patients provided written informed consent. Both trials were performed in accordance with local regulations and approved by the responsible ethics committees (Ethikkommission der Medizinischen Fakultät Göttingen (GLSG1996); Ethikkommission der Medizinischen Fakultät der LMU (GLSG2000). The trials were started before preregistration was implemented and are therefore not registered. LF, LJ, MU, MD and EH analysed the data. All authors had access to all primary data.

### Treatment protocol

CHOP was administered every 3 weeks for 4–6 cycles for patients receiving ASCT and 6–8 cycles for patients receiving IFN-alpha maintenance according to treatment response. Stable disease at end of induction was considered treatment failure and an indication for second line treatment upon the discretion of the investigator. CHOP was administered in standard dosing (intravenous cyclophosphamid 750mg/m^2^, doxorubicine 50mg/m^2^, vincristine 1.4mg/m^2^ (max. 2mg absolute) on day 1 and oral prednisone 100mg/d day 1–5). In the GLSG2000 trial, rituximab was administered at 375mg/m^2^ body surface area on day 0. Stem cell mobilization before ASCT was initiated after cycle 6 with one cycle of dexa-BEAM (intravenous dexamethasone 24mg day 1–10, BCNU 60mg/m^2^ day 2, melphalan 20mg/m^2^ day 3, etoposide 75mg/m^2^ day 4–7, cytarabine 2 × 100mg/m^2^ day 4–7), high dose conditioning consisted of 12 gy TBI and high dose cyclophosphamide (60mg/kg day -3 and -2). IFN was given subcutaneously at 3 × 5mio IU/week or 3mio IU/day (intensified regimen).

Response was assessed after every two cycles of chemotherapy and prior to ASCT. In the first two years after completion of therapy, follow-up was required every three months. Response criteria applied in the trials are in principle consistent with the 1996 International working group criteria (Cheson 1999 [20]; supplement information).

### Outcome

Primary endpoints in this analysis were FFS and OS, tested hierarchically. Secondary efficacy endpoints were overall and complete response rates and duration of response (DOR). FFS was defined as time from start of trial registration to stable disease, progression, or death from any cause. OS was the time from trial registration to death from any cause. DOR was defined as time from end of successful induction (CR, PR) to progression or death from any cause. Safety end points were cumulative incidences of haematological (SHM) and non-hematological (NHSM) secondary malignancies.

### Statistical methods

FFS and OS were described by Kaplan–Meier estimates and compared by log-rank test. For FFS, patients with missing response were censored 1 day after registration, and patients with no treatment failure were censored at last contact in remission. OS was censored at last day of follow-up for patients alive at last contact. A power calculation based on the number of available events in a 2-sided log-rank test with significance level of 5% was performed for both FFS and OS. Based on the 345 available events for FFS and 281 available events for OS and considering a significance level of 5%, this study achieved a power of 80% and 90% to detect clinically relevant hazard ratios for R-CHOP vs. CHOP of 0.74 and 0.71 for FFS and 0.72 and 0.68 for OS, respectively.

Median follow-up was calculated using reverse Kaplan–Meier method. Hazard ratios with 95% confidence intervals and the corresponding p-values were calculated for both univariate and multivariate Cox proportional hazards models adjusted for MIPI continuous score. The primary confirmatory hypothesis tests were performed hierarchically for FFS (first) and OS (second) with two-sided significance level 5% using Cox regression adjusted for MIPI score. Due to hierarchical testing, no adjustment for multiple testing was needed. All secondary statistical tests used a two-sided 5% significance level and were interpreted as hypothesis generating if statistical power was not achieved to show clinically relevant differences.

Overall (ORR) and complete response (CR) rates were compared by Fisher’s exact test. In patients without progression, DOR was censored at last contact in remission. OS after first treatment failure was censored at latest follow-up date for patients alive at last contact. Time-to-event variables were described by Kaplan–Meier estimates and compared by log-rank test. Both unadjusted and adjusted (for MIPI score) Cox regression models were applied to estimate hazard ratios with 95% CI and p values.

Subgroup analyses for FFS and OS were performed according to sex (female; male), MIPI (low; intermediate; high), Ki-67 (< 30%; ≥ 30%), cytology (pleomorphic blastoid or blastic blastoid; non-blastoid) and eligibility for high-dose treatment. For subgroup analyses, potential interaction effects between treatment assignment and subgroup indicators on FFS and OS were explored in multiple Cox regression models. Hazard ratios with 95% CIs calculated from MIPI-adjusted Cox regression models for all the subgroups were displayed in forest plots.

The effect of different second line treatment on OS after first treatment failure was evaluated using Kaplan–Meier estimates, log-rank test and Cox regression models, stratified by with/without Rituximab in second line treatment, and stratified by types of second line treatment (standard chemotherapy with rituximab, standard chemotherapy without rituximab, intensive or high-dose cytarabine containing (immuno-)chemotherapy, ASCT, AlloSCT and others (immunotherapy mono, local therapy, no or unknown)), respectively. Subgroup analyses for second line treatment were performed in treatment assignment (CHOP, R-CHOP) and age at first treatment failure (< 65, ≥ 65) subgroups.

All analyses were performed according to the intention to treat including all MCL patients assigned to CHOP or R-CHOP without censoring for protocol violations. Since this is a pooled analysis and not a randomized comparison, all calculations were also adjusted for MIPI score. Additionally, a post-hoc, exploratory per-protocol sensitivity analysis for FFS and OS was performed, including only patients that started the assigned treatment. Additional adjustment for Ki-67 was not performed, because of missing Ki-67 values in a substantial subset of patients.

The cumulative incidence of treatment failure, death without treatment failure, secondary haematological and non-haematological malignancies were calculated using cumulative incidence function and compared by Gray’s test, treating death without treatment failure, treatment failure, death without a secondary hematological malignancy, and death without a secondary non-hematological malignancy as competing events, respectively.

Statistical analyses were performed using R, version 4.0.4 (www.r-project.org).

## Results

**Fig. 1:**
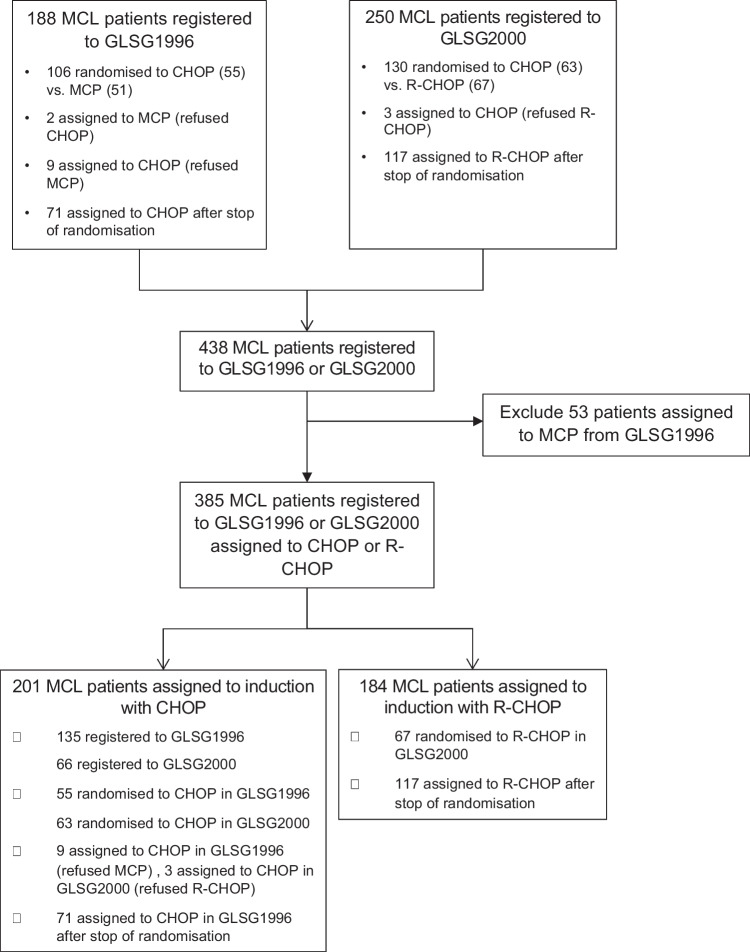
Consort diagram

From May 1996 to May 2003, a total of 438 MCL patients were registered in both trials. 53 patients were assigned to MCP within GLSG1996 and were excluded from this analysis. Therefore, a total of 385 patients were included with *n* = 201 for CHOP and *n* = 184 for R-CHOP (Fig. 1). Key patient characteristics were well balanced between treatment groups (Table 1). Of note, high risk MIPI, Ki67 ≥ 30% and blastoid morphology were present in 21%, 17% and 8% of both cohorts. In the CHOP cohort, 195 patients received CHOP, 2 MCP, 1 R-CHOP and 1 CHVP (cyclophosphamide, doxorubicine, etoposide, prednisolone). In the R-CHOP cohort, 179 patients received R-CHOP and 3 CHOP. 15% and 21% of patients were treated with ASCT in the CHOP and R-CHOP cohort, respectively.

**Table 1:** Patient characteristics

Variable	Value	CHOP (*n* = 201)		R-CHOP (*n* = 184)		*p*
Age (years)	Median, Min–Max	61	37–86	63	35–84	0.46
Sex	Male (*n*, %)	145	72%	146	79%	0.12
Stage	I (*n*, %)	0	0%	2	1% (*n* = 181)	0.53
	II (*n*, %)	1	0% (*n* = 200)	2	1% (*n* = 181)	
	III (*n*, %)	37	18% (*n* = 200)	35	19% (*n* = 181)	
	IV (*n*, %)	162	81% (*n* = 200)	142	78% (*n* = 181)	
ECOG	0 (*n*, %)	59	30% (*n* = 200)	69	38% (*n* = 181)	0.12
	1 (*n*, %)	127	64% (*n* = 200)	98	54% (*n* = 181)	
	2 (*n*, %)	12	6% (*n* = 200)	14	8% (*n* = 181)	
	3 (*n*, %)	2	1% (*n* = 200)	0	0% (*n* = 181)	
LDH (ULN)	Median, Min–Max	0.84	0.15–5.30 (*n* = 200)	0.88	0.44–9.60 (*n* = 179)	0.43
LDH	> ULN (*n*, %)	62	31% (*n* = 200)	65	36% (*n* = 179)	0.28
WBC (G/L)	Median, Min–Max	8.0	1.1–764 (*n* = 197)	7.7	2.7–140.7 (*n* = 179)	0.30
Ki-67	Median, Min–Max	15.1	1.4–90.9 (*n* = 115)	14.3	1.2–91.0 (*n* = 96)	0.86
Ki-67	> = 30%	19	17% (*n* = 115)	16	17% (*n* = 96)	> 0.99
Cytology	Blastoid	5	8% (*n* = 61)	5	8% (*n* = 60)	> 0.99
MIPI score	Median, Min–Max	5.78	4.52–9.18 (*n* = 197)	5.80	4.59–8.60 (*n* = 175)	0.53
MIPI	Low (*n*, %)	84	43% (*n* = 197)	73	42% (*n* = 175)	0.99
	Intermediate (*n*, %)	71	36% (*n* = 197)	65	37% (*n* = 175)	
	High (*n*, %)	42	21% (*n* = 197)	37	21% (*n* = 175)	
Induction treatment started	CHOP (*n*, %)	195	98% (*n* = 199)	3	2% (*n* = 182)	n.a
	R-CHOP (*n*, %)	1	1% (*n* = 199)	179	98% (*n* = 182)	
	CHVP (*n*, %)	1	1% (*n* = 199)	0	0% (*n* = 182)	
	MCP (*n*, %)	2	1% (*n* = 199)	0	0% (*n* = 182)	
Post-remission treatment randomized	ASCT (*n*, %)	40	40% (*n* = 100)	47	49% (*n* = 95)	0.20
	IFN (*n*, %)	60	60% (*n* = 100)	48	51% (*n* = 95)	
Post-remission treatment started	ASCT (*n*, %)	31	15%	39	21%	0.15
	IFN (*n*, %)	110	57% (*n* = 193)	92	51% (*n* = 182)	0.22
	Consolidation (*n*, %)	90	45% (*n* = 199)	91	50% (*n* = 182)	0.36

Patient characteristics of the full cohort of included patients (*n* = 385) stratified by treatment arm (CHOP versus R-CHOP). *ASCT* Autologous stem cell transplantation, *CHOP* Cyclophosphamide doxorubicine vincristine prednisone, *CHVP* Cyclophosphamide doxorubicine vinblastine prednisone, *IFN* Interferon alpha, *MCP* Mitoxantrone chlorambucil prednisone, *MIPI* Mantle cell lymphoma international prognostic index, *R* Rituximab

In line with the primary publication, response rates were higher for R-CHOP in the pooled patient cohort: ORR was 80% vs. 91% (*p* = 0.0032; CR-rate 15% vs. 24%; *p* = 0.037) for CHOP vs. R-CHOP.

**Fig. 2:**
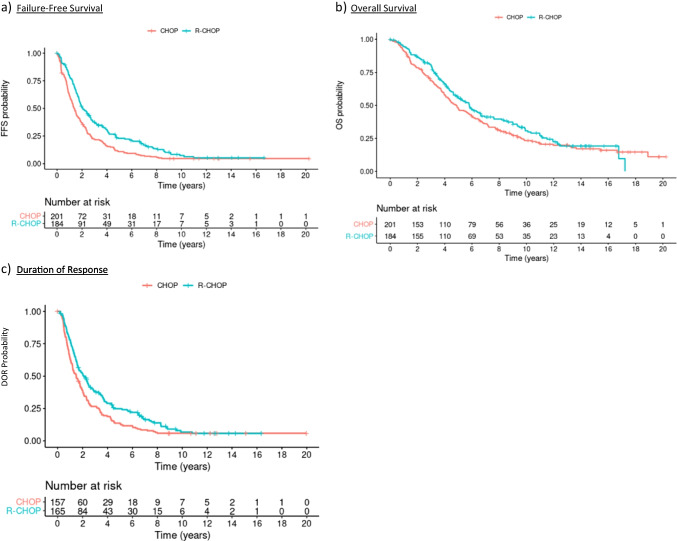
Kaplan–Meier Analysis of Patients treated with CHOP versus R-CHOP. **a** Failure free Survival for patients treated with CHOP versus R-CHOP. Median FFS 1.36 (1.18 – 1.66) vs. 2.07 (1.78 – 2.65) years; 5 year FFS probability 0.11 (0.07 – 0.16) vs. 0.23 (0.17 – 0.30). MIPI-adjusted HR 0.62 (0.50 – 0.77). **b** Overall Survival for patients treated with CHOP versus R-CHOP. Median OS 4.84 (4.10–5.97) vs. 5.81 (4.89–6.94) years; 5 year OS probability 0.48 (0.41–0.55) vs. 0.55 (0.48–0.63); 10-year OS probability 0.23 (0.18–0.30) vs. 0.31 (0.24–0.39). MIPI-adjusted HR 0.78 (0.61 – 0.99). **c** Duration of response for patients treated with CHOP versus R-CHOP. Median DOR 1.48 (1.19 – 1.85) vs. 2.08 (1.65 – 2.65) years; 5-year DOR probability 0.14 (0.09 – 0.20) vs. 0.25 (0.19 – 0.33). MIPI-adjusted HR 0.67 (0.53 – 0.86)

R-CHOP resulted in significantly increased median FFS and OS in the ITT cohort with 1.36 (95% CI: 1.18–1.66) vs. 2.07 (1.78–2.65) years (MIPI-adjusted hazard ratio (aHR) 0.62 (0.50–0.77), *p* < 0.0001) for FFS and 4.84 (4.10–5.97) vs. 5.81 (4.89–6.94) years (aHR 0.78 (0.61–0.99), *p* = 0.039) for OS for CHOP vs. R-CHOP (Fig. 2b-c). The exploratory, post-hoc per-protocol analysis yielded similar results (Fig. S1). Median DOR increased from 1.48 (95% CI 1.19–1.85) to 2.08 (1,65–2,65) years; aHR 0.67 (0.53–0.86; *p* = 0.0012; Fig. 2a).

**Fig. 3:**
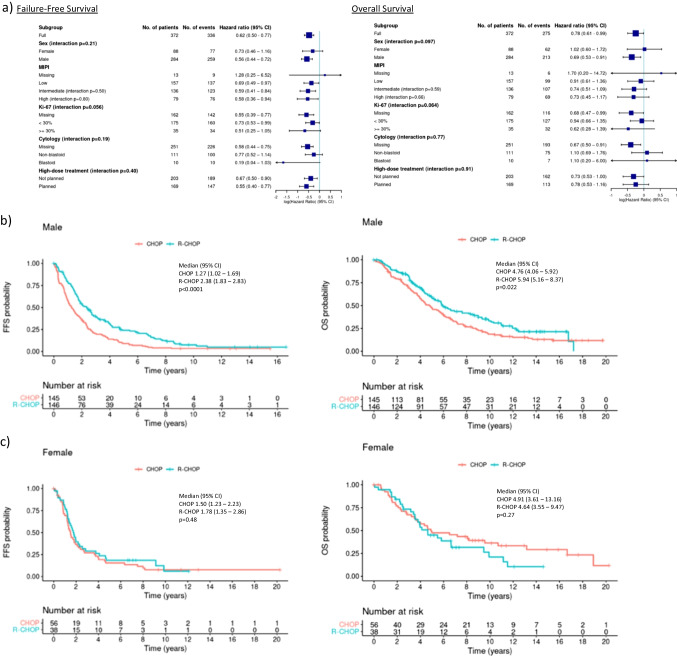
Subgroup analysis for failure free and overall survival. **a** Forest plots for failure free and overall survival. **b** CHOP versus R-CHOP in male patients (*n* = 291). MIPI-adjusted HR for FFS 0.56 (0.44 – 0.72) and for OS 0.69 (95% CI: 0.53 – 0.91). **c** CHOP versus R-CHOP in female patients (*n* = 94), MIPI-adjusted HR for FFS 0.73 (0.46 – 1.16) and for OS 1.02 (0.60 – 1.72)

R-CHOP was similarly effective across all tested MCL risk cohorts (Fig. 3a) with a trend towards larger effects for high-risk populations. R-CHOP resulted in a similarly increased FFS in patients eligible (ASCTe) and ineligible (ASCTi) for ASCT (CHOP vs. R-CHOP: ASCTe 1.3 vs. 2.7 years, aHR 0.58 (95% CI; 0.43 – 0.78), ASCTi 1.4 vs. 1.7 years, aHR 0.66 (0.48–0.91), interaction *p* = 0.4). A similar trend towards longer OS was seen across subgroups for common MCL risk cohorts as well as for ASCTe and ASCTi patients (Fig. 3a).

There was a strong dominance of male patients within GLSG1996 and GLSG2000 trials with 72 and 79% male patients in the CHOP and R-CHOP cohort, respectively. Rituximab demonstrated high efficacy in male patients. Median FFS was increased from 1.27 to 2.38 years (aHR 0.56 (0.44–0.72), *p* < 0.0001). Median OS increased from 4.76 to 5.94 years (aHR 0.69 (0.53–0.91); *p* = 0.0088) (Fig. 3). In the female cohort, FFS and OS differences for CHOP vs. R-CHOP were less pronounced (FFS: 1.50 vs. 1.78 years; aHR 0.73 (0.46–1.16); *p* = 0,18; OS: 4.91 vs. 4.64 years; aHR 1.02 (0.60–1.72); *p* = 0.95) (Fig. 3). The interaction test for treatment outcome according to sex resulted in *p* = 0.21 for FFS and *p* = 0.097 for OS. When adjusted for MIPI and body surface area, hazard ratios in female patients were 0.73 (0.46–1.16, *p* = 0.18) for FFS and 0.98 (0.58–1.67, *p* = 0.95) for OS.

To check for confounders, we looked at possible differences in age, post-remission therapy, number of applied cycles of induction chemotherapy, body-surface, body-mass index, and MIPI score. Apart from body surface, which was lower for female patients, no clinically meaningful differences were seen. Importantly, no difference was seen in body-mass index for women versus men (25 (16–44) vs. 26 (18–40) kg/m^2^). Numerically, women less frequently proceeded to ASCT than men (14.9% vs 19.2%) and accordingly received more cycles of chemotherapy (median 7 vs. 6). Median MIPI was 5.86 (range: 4.71–9.18) for women and 5.77 (4.52–8.60) for men. Comparing women in the CHOP (*n* = 56) or R-CHOP (*n* = 38) cohort, clinical characteristics were similar according to median age (63 years vs. 65 years), BMI (26kg/m^2^ (16–44) vs 25kg/m^2^ (17–38)), ASCT (13% vs 18%), median number of cycles (7.5 vs. 6) and median MIPI (5.78 and 5.95), respectively.

Long-term toxicity: 1 patient in the CHOP and 6 in the R-CHOP cohort developed a SHM, with a cumulative incidence after 10-years of 0.5% and 3.9%. Specifically, we observed 1 myelodysplastic syndrome in the CHOP cohort, 5 MDS and 2 acute myeloid leukaemias in the R-CHOP cohort. The cumulative incidence for SNHM after 10 years was 7 and 8% (16 and 13 cases) for CHOP and R-CHOP, respectively (*p* = 0.92). 2 of 7 patients with SHM and 6 of 29 patients with SNHM received prior ASCT.

**Fig. 4:**
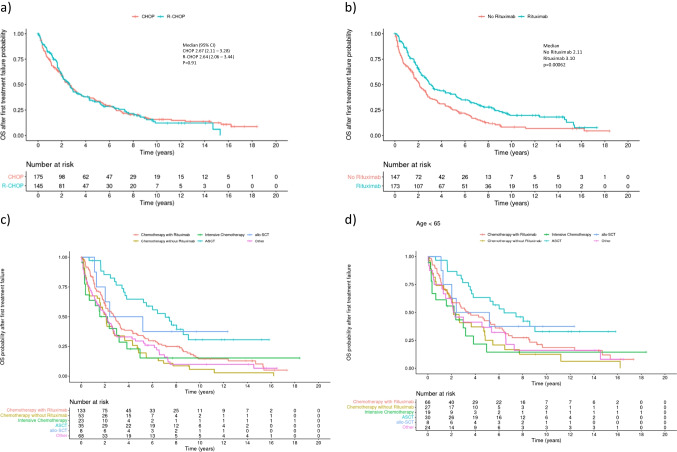
Overall survival after first treatment failure. **a** OS stratified by first line treatment. No significant difference is observed for patients pre-treated with CHOP versus R-CHOP, HR (ref. CHOP) 1.09 (0.83 – 1.41)*, *p* = 0.54*. **b** OS stratified by second line treatment with or without rituximab. HR (ref. no rituximab) 0.70 (0.54—0.91)*, *p* = 0.0077*. **c** OS after first treatment failure stratified by second line treatment for all patients and d) for patients < 65 years. *adjusted for MIPI continuous score, time from registration to first treatment failure, first line treatment

Among all 320 patients alive at first treatment failure (CHOP *n* = 175, R-CHOP *n* = 145), no difference was seen between patients in the CHOP and R-CHOP cohort in terms of OS after first treatment failure (Fig. 4a). 173 (54%) patients received a rituximab-based regimen in second line (47% of patients in the CHOP cohort and 63% in the R-CHOP cohort). Median OS for patients with/without second line rituximab were 3.10 vs. 2.11 years (aHR 0.70 (0.54–0.91), *p* = 0.0077; adjusted for MIPI score, time to first treatment failure and assigned first-line treatment, Fig. 4b). Importantly, this difference was observed as well in the subgroup pretreated with rituximab (aHR 0.55 (0.37–0.83); *p* = 0.0038).

Second line therapies were further grouped into five subgroups to evaluate outcome differences: conventional chemotherapy (*n* = 53), rituximab + conventional chemotherapy (*n* = 133), intensive and/or high-dose AraC containing (immuno-)chemotherapy (*n* = 23), autologous (ASCT, *n* = 35) or allogeneic (alloSCT, *n* = 8) stem cell transplantation. 68 patients received a second line therapy not fitting into these categories: immunotherapy alone (*n* = 21), radiation (*n* = 17), surgery (*n* = 1), no second line therapy (*n* = 25) or second line therapy was unknown (*n* = 4). Only few received targeted second line treatments: One patient received ibrutinib, one patient received temsirolimus, two patients received blinatumomab and one patient received apolizumab, a HLA-DR-ß monoclonal antibody in second line. Median OS was longest for the 35 patients treated with ASCT (7.1 years (95% CI 5.2-NA) in second line, followed by allo SCT and rituximab + conventional chemotherapy (median OS 3.8 and 2.8 years; Fig. 4c). Importantly, when stratified by age at treatment failure, ASCT was still the most effective treatment for patients < 65 years with a median OS of 6.4 years for ASCT (*n* = 30, 95% CI 3.8-NA years) and 3.4 years (*n* = 66, 2.1–6.3 years) for R + conventional chemotherapy, HR 0.57 (0.34–0.96), *p* = 0.035; Fig. 4d).

## Discussion

Evidence regarding OS for the use of rituximab as part of induction immunochemotherapy in previously untreated, advanced stage MCL remains conflicting. To increase statistical power and to perform subgroup analyses, we pooled data of all patients assigned to CHOP or R-CHOP within the GLSG trials 1996 and 2000. To our knowledge, this is the largest cohort of homogeneously treated MCL patients with a standardized follow-up comparing R-chemotherapy to chemotherapy alone. Inclusion criteria and treatment protocols of both trials were virtually identical and both groups were similar in size and well balanced for key patient characteristics. In addition, there were no major differences in post-remission treatments. To further minimize bias, hazard ratios were calculated with adjustment for MIPI score. However, similar results were observed in the unadjusted results (Supplemental Table S1).

With a median follow-up of 13.4 years, we were able to demonstrate a statistically significant benefit in FFS, OS and DOR for R-CHOP compared to CHOP, adding further evidence to the existing literature and underlining the positive effect of rituximab in first line MCL therapy (Fig. 2). The major limitation of this study is, that the CHOP backbone is no more standard of care especially in younger patients and not all eligible patients received consolidating ASCT. This resulted in a relatively short median FFS for both treatment arms in comparison to currently utilized regimens [8, 21, 22]. On the other hand, since rituximab has become part of standard care in MCL, randomized trials investigating the efficacy of rituximab combined with chemotherapy have not been performed.

In exploratory, post-hoc, subgroup analyses we confirm efficacy of rituximab in both, ASCT eligible and ineligible patients as well as across all major MCL subsets, with a trend towards greater benefit for high risk cohorts such as MIPI high risk and Ki67 proliferation index ≥ 30% (Fig. 3a). This observation is in line with the guideline recommendations to add rituximab in both patient groups.

Secondly, efficacy of rituximab in our study was especially pronounced in male MCL patients (Fig. 3). Generally, only 1 in 3–4 of all MCL patients are female [3, 4]. So far the underlying biological cause for the observed male dominance in MCL remains unclear, but the 4:1 ratio lead some authors to propose a role of sex chromosome linked genetic or epigenetic alterations as a possible factor [23]. Our analysis of sex-specific outcome is hampered by several possible confounders: The female cohort in our study is expectedly significantly smaller than the male cohort, therefore possibly lacking in statistical power to detect survival differences. In addition, body composition and applied treatments might differ between genders. To account for this potential bias, we analysed differences in age, body surface area (BSA), body mass index (BMI), number of chemotherapy cycles, post-remission treatment and MIPI. However, we did not observe meaningful differences in age, BMI or post-remission treatment. As expected, body-surface area was lower in female patients, but was similar within the female CHOP and R-CHOP cohorts. In addition, female patients in the R-CHOP cohort had a higher median MIPI compared to male patients or female CHOP patients. However, all HRs were adjusting for MIPI score and a second calculation adjusting for both MIPI and body surface area resulted in similar results.

So far published data regarding sex-specific outcome after rituximab is inconsistent: A series of observations, mainly performed in aggressive lymphoma, suggest slower rituximab clearance in elderly female patients, associated with a beneficial effect on response and survival for female compared to male patients [24–26]. In indolent lymphoma, a small pharmacokinetic trial, as well as a single centre retrospective trial suggested, that elderly female patients might have better outcomes with rituximab-based chemotherapies compared to elderly male patients [27, 28]. In contrast to those studies, a post-hoc analysis performed by our study group of 1172 follicular lymphoma (FL) patients prospectively treated within the same multicentre GLSG1996 and 2000 trials, comparing the effect of rituximab in male and female patients, yielded results in line with the observations of this study: Male patients had a significantly worse outcome than female patients when treated with CHOP (*p* = 0.0041), but outcome was similar in the R-CHOP cohorts. Accordingly, efficacy of rituximab was more pronounced in the male cohort (HR: 0.31 (95% CI; 0.21–0.46) vs. 0.53 (0.37–0.76) in female patients) [29]. Additionally, a recently published analysis of a pooled cohort of CLL-patients implicated, that especially obese female patients may profit less from the addition of rituximab to fludarabine / cyclophosphamide [30].

In conclusion, data on sex specific response to rituximab treatment in lymphoma remains conflicting, warranting further studies tackling possible biological differences. Our data underlines the importance of considering sex differences when applying targeted treatment strategies.

We did not observe clinically meaningful differences in late toxicities. The number of 7 patients developing a SHM is too small to evaluate and SHNM were similar between both cohorts.

In an additional exploratory descriptive post hoc analysis, we calculated OS outcomes for patients who relapsed after first line therapy, stratified by second line treatment and age at relapse. Firstly, we did not observe survival differences after first relapse in the CHOP versus R-CHOP arm, indicating that the OS benefit results from prolonged first remissions in the R-CHOP arm (Fig. 4a). Additionally, patients receiving rituximab in first relapse had a significantly longer OS compared to other patients with a HR of 0.7 and a median OS of 3.1 years (Fig. 4b). Importantly, this effect was confirmed in the whole cohort, as well as the subset of R-CHOP patients, indicating that rituximab retreatment is effective.

The best outcome after second line treatment was observed for ASCT with a median OS of 7.1 years. Importantly, this was still true, if patients were stratified by age at relapse, with a median OS of 6.4 vs. 3.4 years for younger patients after ASCT vs. R-chemotherapy, respectively. However, as a confounding factor, the ASCT cohort consists presumably of fit patients with chemotherapy sensitive relapses as indicated by response to salvage therapies.

In general, the OS after treatment failure of this cohort of patients aged 35–86 years and recruited between 1996 and 2003, was comparable even to more recently published cohorts: In a pooled analysis of 3 trials investigating ibrutinib, 3-year OS in second line patients was around 60% [31]. Retrospective real-world cohorts report median OS times of 24–36 months after first relapse [32, 33]. An analysis of younger patients treated within the Nordic MCL2 and MCL3 trials found a median OS for early relapsed patients (POD24) of 6.6 months versus 46 months for patients relapsing after 24 months [34].

In summary, our data underlines, that prolonging remissions after first line treatment is of paramount importance to improve the long term outcome of the disease whereas in relapsed MCL there is an urgent medical need for further improvement of salvage approaches.

In conclusion, the CD20 monoclonal antibody Rituximab is effective in previously untreated, advanced stage mantle cell lymphoma if added to standard CHOP chemotherapy, resulting in a prolonged FFS, OS and DOR. Rituximab is furthermore effective across different MCL risk groups in high as well as in low-risk patients and in first as well as in later lines of therapy. Therefore, rituximab should remain part of first-line treatment of MCL unless new evidence becomes available.

## Supplementary Information

Below is the link to the electronic supplementary material.

Supplementary file1 (PDF 132 KB)

## Data Availability

Anonymized clinical data underlying the analysis might be shared upon request to the corresponding author on the basis of scientific collaboration.
