# Supplementary material for: The addition of rituximab to chemotherapy improves overall survival in mantle cell lymphoma—a pooled trials analysis
Source: Ann Hematol. 2023 Aug 8;102(10):2791–801. doi: 10.1007/s00277-023-05385-1 (PMC10492741; doi:10.1007/s00277-023-05385-1)
Supplement: Supplementary file 1 — Supplementary file1 (PDF 132 KB) [file 277_2023_5385_MOESM1_ESM.pdf]

Supplemental material

Supplemental methods:

Response assessment: Complete remission (CR) was defined as complete disappearance of all previously identified lymphadenopathies, hepato- / splenomegaly and bone marrow involvement. Partial remission (PR) was defined as ≥ 50 % decrease in size of previously identified lymphoma manifestations without new lymphoma lesions. Progression was defined as an increase of 25 % in volume of lymphoma lesions, appearance of new lymphoma lesions, as well as worsening of lymphoma associated symptoms.

Endpoints: FFS is defined as time from start of trial registration to stable disease, progression, or death from any cause. OS is the time from trial registration to death from any cause. DOR is defined as time from end of successful induction (CR, PR) to progression or death from any cause. To distinguish different event types in the combined endpoint FFS, cumulative incidence of treatment failure and cumulative incidence of death without treatment failure were also evaluated. As only long-term safety endpoints, we estimated cumulative incidences of haematological and non-haematological secondary malignancies. Finally, to explore outcomes after first treatment failure, OS from first treatment failure was described, with and without stratification by type of second line treatment.

Supplemental Tables:

Table S1 Unadjusted Analysis

	**Hazard ratio**	**95% Confidence Interval**	**p-Value**
Survival for CHOP vs. R-CHOP in the whole cohort:			
**DOR**	0.73	0.58 – 0.92	0.0070
**FFS**	0.66	0.54 – 0.82	0.00016
**OS**	0.85	0.67 – 1.08	0.18
**OS after 1st treatm. Failure**	1.01	0.79 – 1.30	0.91
FFS for CHOP vs. R-CHOP in subgroup analysis:			
**Ki67 <30%**	0.76	0.55 – 1.03	0.077
**Ki67 ≥30%**	0.48	0.24 – 0.97	0.042
**Non-blastoid morphology**	0.73	0.49 – 1.09	0.12
**Blastoid morphology**	0.26	0.06 – 1.13	0.073
**Male sex**	0.60	0.47 – 0.76	0.0001
**Female sex**	0.85	0.55 – 1.33	0.48

Supplemental Tab.S1 *Hazard ratios for the unadjusted analysis of CHOP vs. R-CHOP.*

Supplemental Figures:

Figure S1

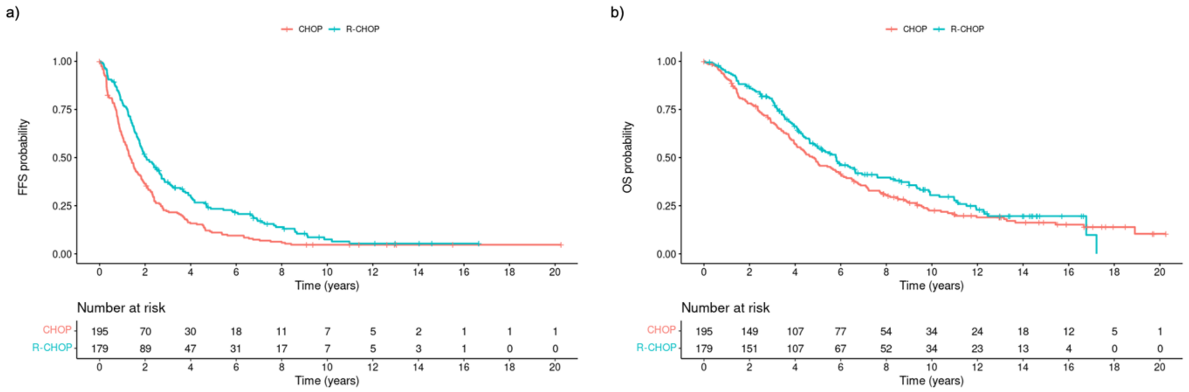

Fig.S1 Kaplan-Meier Analysis of Patients treated with CHOP versus R-CHOP, per-protocol sensitivity analysis a) Failure free Survival for patients treated with CHOP versus R-CHOP. Median FFS 1.3 (1.1 – 1.7) vs. 2.1 (1.8 – 2.6) years. MIPI-adjusted HR 0.62 (0.50 – 0.78). b) Overall Survival for patients treated with CHOP versus R-CHOP. Median OS 4.8 (4.1-5.9) vs. 5.8 (4.8-6.9) years. MIPI-adjusted HR 0.78 (0.61 – 0.99)
